# Associating with kin selects for disease resistance and against tolerance

**DOI:** 10.1098/rspb.2024.0356

**Published:** 2024-05-22

**Authors:** Jason C. Walsman, Madalyn Lambe, Jessica F. Stephenson

**Affiliations:** ^1^ Department of Biological Sciences, University of Pittsburgh, Pittsburgh, PA, USA; ^2^ Earth Research Institute, University of California-Santa Barbara, Santa Barbara, CA, USA

**Keywords:** behavioural immunity, resistance evolution, kin selection, physiological immunity, infectious contacts, infection transmission

## Abstract

Behavioural and physiological resistance are key to slowing epidemic spread. We explore the evolutionary and epidemic consequences of their different costs for the evolution of tolerance that trades off with resistance. Behavioural resistance affects social cohesion, with associated group-level costs, while the cost of physiological resistance accrues only to the individual. Further, resistance, and the associated reduction in transmission, benefit susceptible hosts directly, whereas infected hosts only benefit indirectly, by reducing transmission to kin. We therefore model the coevolution of transmission-reducing resistance expressed in susceptible hosts with resistance expressed in infected hosts, as a function of kin association, and analyse the effect on population-level outcomes. Using parameter values for guppies, *Poecilia reticulata*, and their gyrodactylid parasites, we find that: (1) either susceptible or infected hosts should invest heavily in resistance, but not both; (2) kin association drives investment in physiological resistance more strongly than in behavioural resistance; and (3) even weak levels of kin association can favour altruistic infected hosts that invest heavily in resistance (versus selfish tolerance), eliminating parasites. Overall, our finding that weak kin association affects the coevolution of infected and susceptible investment in both behavioural and physiological resistance suggests that kin selection may affect disease dynamics across systems.

## 1. Introduction

Hosts employ multiple defences to prevent parasite transmission (encompassing parasites and pathogens [1–3]), and these defences differ in their costs and benefits. For example, transmission-reducing, asocial behaviour (which can be host-driven behavioural resistance) is likely to have costs as asocial behaviour can lead to loss of social benefits in many species [4,5]; these costs are probably experienced by the focal, asocial individual and possibly also by non-focal individuals missing out on the benefits of social contact (e.g. individuals in smaller groups have lower fitness in various social mammal species [6]). Physiological immune investment (at times referred to as ‘tissue-specific’ [7]), on the other hand, acts within the body of an individual host to limit parasite growth and/or prevent transmission and often carries internal, physiological costs borne only by the focal individual expressing the resistance [8–11]. Costs of behavioural or physiological resistance can be borne by susceptible hosts when they express resistance to physiologically prevent infection or behaviourally avoid infected hosts; costs can also be borne by infected hosts, e.g. costly self-isolation (behavioural resistance) or costly reduction of parasite shedding (physiological resistance). However, the transmission-reduction fitness benefits of resistance accrue differently to susceptible hosts and those already infected: while susceptible hosts directly benefit from their resistance that prevents their own infection, infected hosts only benefit indirectly, by reducing transmission to kin.

**Figure 1.**
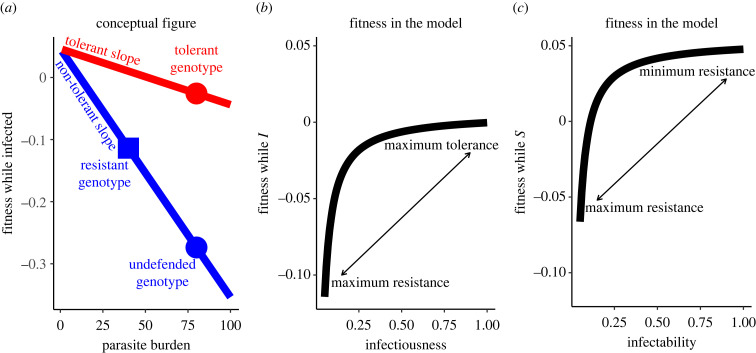
Resistance, tolerance and the fitness costs of reducing transmission. (*a*) Two core physiological immune defences, resistance and tolerance, are often defined with respect to parasite burden and fitness while infected. Compared to an undefended genotype (blue circle), a resistant genotype reduces parasite burden on each infected host, thus increasing host fitness while infected and decreasing parasite fitness (blue square). By contrast, tolerant genotypes (red circle) do not reduce parasite burden and thus do not reduce parasite fitness but increase host fitness while infected. Tolerant genotypes accomplish this by reducing the impact of each unit of parasite burden on host fitness (red, tolerant slope is shallower than blue, non-tolerant slope). Panel (*a*) is conceptual as we do not explicitly model within-host infection dynamics such as parasite burden. (*b*) More tolerant host genotypes, as they do not affect parasite fitness, tend to have higher parasite burdens and thus higher infectiousness, as well as maintaining higher fitness while infected (*I*), because we here assume tolerance is more efficacious than resistance in terms of direct fitness. Compared to more tolerant genotypes, more resistant, less infectious genotypes have lower fitness while infected. (*c*) Fitness while susceptible (*S*) also increases with infectability in our model, as constitutive resistance to becoming infected bears a fitness cost. Thus, both resistance to becoming infected (reducing infectability) and resistance while infected (reducing infectiousness), have direct fitness costs in our model. Panels (*b*) and (*c*) reflect the functional forms and parameters we use for physiological resistance.

This topic touches on critical, ongoing questions of when hosts should evolve resistance instead of or in addition to tolerance and how such evolution will impact population-level outcomes. Particularly, selection may favour resistance over tolerance when spatial structure leads to kin association [12], assuming proximity matters for transmission; these results should interact with whether the costs of resistance are borne by the individual (physiological resistance) or by the group (as may arise in behavioural resistance). While much previous work has focused on physiological resistance or tolerance, a growing body of work emphasizes the importance of behavioural resistance or behavioural tolerance [7,13,14]. Studies vary widely in how (or whether) they define ‘resistance’ and ‘tolerance’. We focus on ‘resistance’ as a host trait that directly lowers parasite fitness while ‘tolerance’ is a host trait that does not lower parasite fitness but maintains higher host fitness given the same infection (following the definition of [15]). We give a conceptual example of how physiological resistance and tolerance are sometimes measured in figure 1*a* as well as the actual values and functional forms we use in our model in figure 1*b,c*. Infected hosts often face a trade-off between these forms of host defence (empirically demonstrated for physiological host defence [16–19] and with some support in behavioural host defence [13,20,21]). Further, there is no general answer for whether tolerance or resistance will improve host fitness most [15]. We choose a tolerance–resistance trade-off in which infected resistance does not improve health as much as tolerance does (shown in figure 1*a,b*), as supported by some empirical evidence for physiological [16,17] and behavioural resistance [20,21]. Thus, we explore the scenario in which resistance by infected hosts only makes sense through the benefits of not transmitting the infection to kin; otherwise, selection favours tolerance.

In addition to the trade-off between resistance and tolerance in infected hosts, we consider a trade-off in the traits of susceptible hosts. As found in diverse systems [22–24], susceptible hosts can reduce their risk of infection (e.g. invest in resistance to infection), but with a cost borne in terms of their fitness while susceptible (shown for physiological resistance in figure 1*c*). Resistance that prevents infection has often been termed ‘avoidance’ (e.g. [19]), but we attempt to clarify physiological versus behavioural mechanisms. We term physiological resistance by susceptible hosts as reduced ‘infectability’ while physiological resistance by infected hosts is reduced ‘infectiousness’. We use reduced ‘gregariousness’ to mean behavioural resistance by both susceptible and infected hosts, even though reducing gregariousness will not directly change the infection status of the infected individual. We consider how the coevolutionary interplay between resistance by susceptible and resistance versus tolerance by infected hosts depends on whether we model behavioural or physiological resistance.

For both models, infected hosts should only invest in costly resistance if kin selection is very important relative to selection on direct fitness [25,26]. This has been empirically observed as self-isolation (behavioural resistance) in eusocial insects, in which kin association is very frequent and thus kin selection should be extremely important [20,21,27]. However, investigation of active self-isolation is lacking in other systems [26], potentially because it can be difficult to disentangle when self-isolation is beneficial for the infected host's direct fitness [28]. For physiological resistance, some previous modelling has shown that kin association can favour transmission-blocking by infected hosts [12] (these models have individual costs [29]). An extreme form of such physiological resistance, suicide by infected hosts, evolved in clonal bacterial populations with spatially driven kin association [30]. However many studies, theoretical and empirical, have assumed kin association is usually too weak to matter for the evolution of altruistic resistance by infected hosts (we use ‘altruistic’ to refer to a trait that reduces direct fitness and increases the fitness of other individuals, following Hamilton [31]). Instead, much previous work has either ignored evolution of infected resistance or considered only selection on direct fitness for infected hosts. In nature, however, kin association falls across a broad range (e.g. with estimates of within-group relatedness ranging from nearly 0 to 0.78 across vertebrate taxa [32–35]). So how strong does kin association need to be for altruistic infected resistance to evolve?

This question of infected resistance cannot be adequately addressed without considering the feedbacks between the evolution of infected resistance and susceptible resistance. If infected hosts evolve more or less resistance (behavioural or physiological), then susceptible hosts may as well; and resistance investment by either class may increase or decrease selection for resistance investment by the other class. Such differential investment in resistance has received particular empirical attention for behavioural resistance. Infected hosts have expressed lower gregariousness [higher behavioural resistance; 20,21,36] or higher gregariousness [37,38] than susceptible hosts, suggesting these may entail two traits that can coevolve. For physiological resistance, the infectability of susceptible hosts and infectiousness of infected hosts are quite likely to be different traits and thus capable of coevolving. Little of the existing intuition and modelling have considered interactions between susceptible immune investment and infected immune investment, particularly in regards to kin selection (as noted by Lion & Gandon [29]).

Lastly, how do these questions apply to the cases of behavioural and physiological resistance? Long-standing and sustained attention has been given to understanding the action and relative importance of behavioural and physiological host defence [1,3,39–43], particularly because they can govern transmission rates in similar ways. However, very little theoretical work has directly compared and contrasted the evolution of these defences [44]. We are unaware of any that consider kin selection, or the shared costs of behavioural resistance versus the individual costs of physiological resistance. Thus, kin selection may interact differently with the coevolution of resistance in the behavioural resistance case and the physiological resistance case.

We explore how the form of resistance, kin association, and coevolution of susceptible and infected hosts alter selection for resistance versus tolerance with a biologically reasonable model. We use parameter values [45] from a guppy-worm system (*Poecilia reticulata*-*Gyrodactylus* spp*.*) with social transmission [46] and heritable variation in behavioural resistance governing contact [47,48] and physiological resistance [49,50]. Guppies may associate with kin as juveniles in the laboratory [51] and the wild [33] but they do not as adults [51,52]. Further, uninfected and infected hosts can differ in their behavioural resistance [3] as infected guppies may seek contact [3,53] while uninfected guppies may avoid contact [46,54,55], potentially reducing overall rates of social contact [55]. Thus, this focal system provides empirically derived parameter values as well as empirical motivation for model ingredients like weak kin association and infection-class differences in behavioural resistance. To see a range of possible outcomes that may be broadly relevant to various systems, we consider a range of kin associations from none to very strong. We compare and contrast results for a model of behavioural versus a model of physiological resistance; for both models, selection for altruistic resistance in infected hosts can occur even when kin association is rare. This result has important implications for infection prevalence and host density at the population level, which may be important across systems for management concerns such as disease spillover or host extinction. Further, the two different models of resistance, coevolution of infected and susceptible hosts, and kin selection all build substantially on our understanding of when resistance or tolerance will evolve.

## 2. Methods

We consider two models (behavioural or physiological) of coevolution of resistance investment by susceptible and infected hosts with a differential equation model and evolutionary invasion analysis. For behavioural or physiological resistance, evolution is represented in the trait *g_X, Y_*, which depends on genotype *Y* and infection status *X* (*S* for susceptible, *I* for infected, or *H* = *S + I*). Higher values of *g_X, Y_* correspond to higher transmission rates given encounter between a susceptible and infected host, i.e. lower resistance, and generally correspond to higher direct fitness for hosts of a given infection status; thus, higher *g_I, Y_* corresponds to higher fitness while infected, lower resistance and disease tolerance (similar to Horns & Hood [12], who model physiological tolerance as higher fitness while infected and higher transmission rate compared to resistance). For the behavioural resistance model, *g_S, Y_* represents the gregariousness of susceptible hosts of genotype *Y* and *g_I, Y_* is the gregariousness infected hosts. Higher gregariousness generally leads to more social contacts for the focal individual (*C_XY_*_, Total_ is the total rate of social contacts for an individual of genotype *Y* and infection status *X* with the rest of the host population) as well as for non-focal individuals; more social contacts lead to lower death rate from non-disease sources, e.g. living in groups helps defend against predation in many systems [56], including the focal guppy system [57] (predation death is *B*/*C_XY_*_, Total_ where *B* scales predation).

For the physiological resistance model, *g_S, Y_* is the infectability of susceptible hosts of genotype *Y* and *g_I, Y_* is the infectiousness of infected hosts; physiological resistance investment lowers *g_X, Y_* and thus transmission but carries a mortality cost borne by the focal individual in terms of increased death rate from lower tolerance, e.g. due to immunopathology [58] (immunopathology death is *B*/*g_X, Y_*). In both models, resistance reduces transmission rate (lower *g_X, Y_*) but carries a cost of increased mortality borne either by the focal individual (physiological resistance) or by the focal individual and non-focal individuals (behavioural resistance).

Resistance reduces the chance of transmission given encounter between an infected and uninfected individual. Each individual has a fixed *E* encounters per day which are divided into random encounters (*R*) with all genotypes at frequency-dependent rates and non-random, assortative encounters that are only with kin (1 − *R*, following the classic model of Eshel & Cavalli-Sforza [59]). Higher rates of random encounters correspond to lower values of kin association. For simplicity, we only consider kin as individuals of the same, clonal genotype. Thus, the function for encounters per time unit experienced by an individual of class *X*_Y_ with individuals of class *W*_Z_ is given by equation (2.1*a*). Note that the *W_Z_* class may or may not be the same class as the *X_Y_* class of the focal individual.

2.1a $$En_{XY,WZ}=\underset{\mathrm{Random}\;\mathrm{contacts}}{\underbrace{ER\frac{W_{Z}}{H_{T}}}}+\underset{\mathrm{Non}-\mathrm{random}\;\mathrm{contacts}}{\underbrace{E(1-R)\delta_{Y,Z}\frac{W_{Z}}{H_{Y}}}}$$

and

2.1b $$\mathrm{Transmission}\;\mathrm{rate}\;\mathrm{from}\;I_{Z}\;\mathrm{to}\;S_{Y}=Tg_{S,Y}g_{I,Z}En_{SY,IZ}$$

Here *δ*_Y, Z_ is the Kronecker delta (1 when *Y* = *Z* and 0 when *Y* ≠ *Z*); thus, individuals have random contacts with kin and non-kin but non-random contacts only occur between individuals of the same genotype (i.e. are kin). Transmission from infected hosts (e.g. of genotype *Z*) to susceptible hosts (e.g. of genotype *Y*) then occurs at a rate directly proportional to encounter rate (equation (2.1*b*)). Transmission rate is also directly proportional to some transmissibility parameter (*T*) and the *g* parameters that depend on resistance of susceptible (*g_S,Y_*) and infected (*g_I,Z_*) hosts. In the behavioural resistance model, *g_X,Y_* is gregariousness and can be thought of as the probability of accepting contact given encounter (ranging from 0 to 1), contributing to the joint probability of both individuals accepting contact gives the contact rate (*C_XY, WZ_* = *g_X,Y,_ g_W,Z_ En_XY, WZ_*); thus, the gregariousness of another individual (*g_W,Z_*) influences the contact rate an individual experiences and thus the mortality cost of resistance investment is shared (e.g. higher *g_W,Z_* can lead to higher *C_XY, WZ_*, thus higher *C_XY_*_, Total_, and thus lower death rate of *X, Y* individuals). The gregariousness of a focal genotype will be more important for the fitness of non-focal genotypes if the focal genotype is very frequent. If instead, the focal genotype has a very low frequency, then there will be very few encounters between the two genotypes (equation (2.1*a*)) and the gregariousness of the focal genotype will have very little impact on the death rate of the non-focal genotype. This mathematical logic could be instantiated, for example, if the non-focal genotype is present in a large group and the single individual of the focal genotype leaves the group due to low gregariousness, having little impact on the fitness of the non-focal genotype.

In the physiological resistance model, these *g* parameters are simply susceptible infectability and infected infectiousness, respectively, and they simply scale transmission rate (also limited to 0–1 for comparability to behavioural resistance). Since each individual has a fixed *E* encounters per day, parasite transmission is frequency-dependent for both models. Frequency-dependent transmission may be reasonable for our focal system [60] and is a necessary assumption or else different levels of kin association would artificially alter parasite transmission (e.g. any rare genotype would suffer essentially zero infection in the case of all non-random, assortative encounters and density-dependent transmission). We embed this function for encounters and parasite transmission within an SI model of host densities.

We model ecological dynamics of the susceptible host density, *S*_r_, and infected host density, *I*_r_, of a resident genotype, r. Host populations grow logistically with maximum fecundity *b* and sensitivity to crowding *q*; both births and crowding depend on the total density of the resident genotype: *H*_r_ = *S*_r_ + *I*_r_ (equation (2.2*a*)). Susceptible hosts die with some background mortality rate *d* and additional mortality reflecting the cost of resistance. Susceptible individuals are also lost to infection depending on transmissibility of infection, *T*, and resistance of susceptible (*g_S,r_*) and infected hosts (*g_I,r_*), and the rate of encounters with infected hosts (*En_S_*_r*,I*r_). Once infected, hosts suffer background mortality and additional mortality related to resistance investment; they also suffer additional mortality due to the virulence, *v*, of infection (equation (2.2*b*)). These equations are very similar for the behavioural resistance model (equation (2.2*a*,*b*)) and the physiological resistance model (equation (2.2*c*,*d*)) except that the cost of resistance depends on resultant total contacts for behavioural resistance (written out for clarity in equations (2.2*a*,*b*)) but simply on resistance for physiological resistance. This functional form for the costs of resistance creates diminishing marginal returns and often selects for intermediate resistance (figure 1*b,c* curvature).

Behavioural resistance:

2.2a $$\frac{dS_{r}}{dt}=(b-qH_{r})H_{r}-dS_{r}-\frac{B}{C_{Sr,Sr}+C_{Sr,Ir}}S_{r}-Tg_{S,r}g_{I,r}En_{Sr,Ir}S_{r}$$

2.2b $$\frac{dI_{r}}{dt}=Tg_{S,r}g_{I,r}En_{Sr,Ir}S_{r}-dI_{r}-vI_{r}-\frac{B}{C_{Ir,Sr}+C_{Ir,Ir}}I_{r}$$

Physiological resistance:

2.2c $$\frac{dS_{r}}{dt}=(b-qH_{r})H_{r}-dS_{r}-\frac{B}{g_{S,r}}S_{r}-Tg_{S,r}g_{I,r}En_{Sr,Ir}S_{r}$$

2.2d $$\frac{dI_{r}}{dt}=Tg_{S,r}g_{I,r}En_{Sr,Ir}S_{r}-dI_{r}-vI_{r}-\frac{B}{g_{I,r}}I_{r}$$

We model evolution through invasion analysis. For simplicity, we do not impose any correlation between an individual's trait when susceptible, *g_S, Y_*, and its trait when infected, *g_I, Y_*, which may match gregariousness in the focal system [3]*.* The dynamics of an invading mutant follow equation (2.3).

Behavioural resistance:

2.3a $$\frac{dS_{m}}{\mathrm{dt}}=(b-qH_{r})H_{m}-dS_{m}-\frac{B}{C_{Sm,Sm}+C_{Sm,Im}+C_{Sm,Sr}+C_{Sm,Ir}}S_{m}-T(g_{S,m}g_{I,m}En_{Sm,Im}+g_{S,m}g_{I,r}En_{Sm,Ir})S_{m}$$

and

2.3b $$\frac{dI_{m}}{dt}=T(g_{S,m}g_{I,m}En_{Sm,Im}+g_{S,m}g_{I,r}En_{Sm,Ir})S_{m}-dI_{m}-vI_{m}-\frac{B}{C_{Im,Sm}+C_{Im,Im}+C_{Im,Sr}+C_{Im,Ir}}I_{m}$$

Physiological resistance:

2.3c $$\frac{dS_{m}}{\mathrm{dt}}=(b-qH_{r})H_{m}-dS_{m}-\frac{B}{g_{S,m}}S_{m}-T(g_{S,m}g_{I,m}En_{Sm,Im}+g_{S,m}g_{I,r}En_{Sm,Ir})S_{m}$$

and

2.3d $$\frac{dI_{m}}{dt}=T(g_{S,m}g_{I,m}En_{Sm,Im}+g_{S,m}g_{I,r}En_{Sm,Ir})S_{m}-dI_{m}-vI_{m}-\frac{B}{g_{I,m}}I_{m}$$

We use parameter values reasonable for the focal system. This system, and thus many of our parameter values, are highly influenced by the prevailing predation pressure: our choice to use values reflecting high predation conditions throughout corresponds to a stronger, gregariousness-based trade-off. In high predation populations (relative to low predation populations), gregariousness is very important for guppies to avoid death from predation; but high predation populations have also been found to have more transmissible and virulent parasites, leading to a higher downside of gregariousness in the risk of infection and death. Most values were previously empirically derived (*b* = 0.106, *q* = 0.017, *d* = 0.0013, *v* = 0.48, *T* = 0.0081 [45]), but two parameter values required our judgement. First, we chose encounter rate (*E* = 50) to lead to reasonable prevalence for high predation populations at intermediate resistance (*g* values around 0.5 and accounting for the fact that our model here does not include host recovery, unlike the focal system). Second, we chose *B* (0.3 for behavioural resistance and 0.006 for physiological resistance) such that hosts reaping the benefits of minimum resistance investment (*g_X, Y_* = 1) experienced the same investment-dependent death rate as that of wild hosts experiencing the lowest, investment-dependent death rate in the previously published parametrization [45]. We use these focal, empirically grounded parameter values so that we can characterize the model behaviour in a parameter space that is reasonably representative of at least one biological system, if not more. We contrast these results with others that may apply for other systems with stronger kin association by varying the *R* parameter from 0 to 1. Lastly, we further tested the robustness of our key results with a Latin hypercube search of 500 parameter value sets with each parameter uniformly distributed within ±50% of the corresponding focal parameter value (except *R* which we varied uniformly from 0 to 1). Thus, the focal parameter set gives us a biologically reasonable center around which to expand our numerical analyses for the purposes of comparing likely results for our focal system and other systems.

We varied the frequency of random encounters widely to capture a range of coevolutionary outcomes that may be representative of various host taxa. For clonally reproducing organisms with very limited dispersal, *R* would be approximately 0 as nearly all encounters are disproportionately with kin, even if kin have low frequency on the landscape. For other species, we can approximate qualitatively reasonable *R* values based on what would produce empirical values of mean within-group relatedness (*r* in the literature). We assume that the vast majority of an animal's social encounters occur with members of its group. Within-group relatedness then gives the probability that another individual has the same allele from a common ancestor which we take as the portion of encounters which are with the same genotype. If a genotype has frequency *f* in the population as a whole, then *r* = *Rf* + (1 − *R*) and this can be re-arranged to *R* = (1 − *r*)*/*(1 − *f*). When possible, we approximate between-group relatedness as *f*, the frequency of the genotype across groups; but in general, we expect *f* to be small in nature and have little impact on the calculation of *R*. For Trinidadian guppy shoals, *r* can be 0.0216 with *f* = 0.0123 giving *R* = 0.99 [33]. For various mammals, *r* can range from 0 to 0.5 [34,35], which, assuming some small *f* such as 0.05, implies *R* from 0.52 to 1. Red-winged blackbird data can imply *R* values as low as 0.3 ((1 – 0.78) / (1 – 0.28) [32]). To capture the biological extremes, we show a spectrum of outcomes from *R* = 0 to 1.

With our empirically reasonable parameter values, we find the trait and population-level outcomes of coevolution. Numerically, we find the stable equilibrium of the resident genotype. Then we simulate a mutant at very low density and determine whether invasion fitness is positive or negative. Then we find a trait value of susceptible resistance at which evolution stops (evolutionary singular point). For a given rate of random encounters, *R*, we find the singular point(s) of susceptible resistance as a function of infected resistance and vice versa. An endpoint of coevolution must be an intersection of the two; further, we check the coevolutionary stability of any such intersection to determine whether populations with trait values in that neighbourhood evolve toward that intersection of trait values and stay there, making that intersection an endpoint of coevolution [61,62]. Lastly, we calculate infection prevalence and host density for single-genotype populations to show how evolution toward a coevolutionary attractor alters population-level outcomes.

## 3. Results

**Figure 2.**
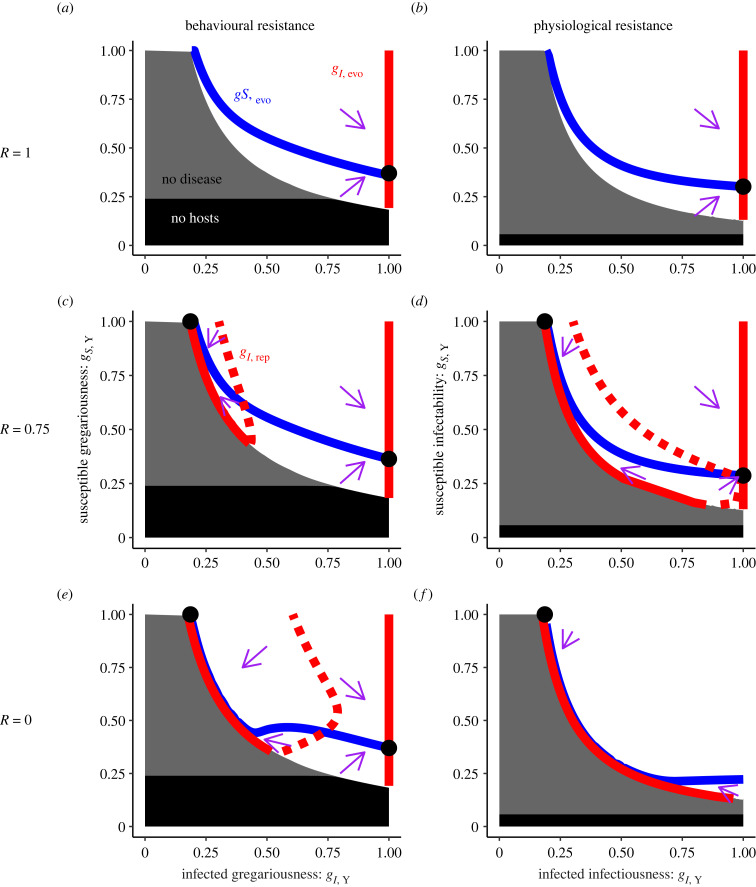
Infected hosts may selfishly spread parasites through tolerance or altruistically block transmission through resistance that protects their kin. We show the phase plane of coevolution of infected and susceptible traits. The first column shows results for the behavioural resistance model while the second shows results for the physiological resistance model. Each row shows results for a different value of the rate of random encounters, *R*. (*a*) If infected and/or susceptible gregariousness are too low (high resistance), the stable equilibrium may have no hosts (black shading) or hosts but no parasites (grey shading). Within the parameter space with a stable, endemic equilibrium (white background), we show susceptible gregariousness that evolves at a given level of infected gregariousness (*g_S,_*_evo_, blue curve), infected gregariousness that evolves as a function of susceptible gregariousness (*g_I,_*_evo,_ red curve), the intersection of the two (black point), and the general direction of coevolution (purple arrows). (*b*) These results are very similar for the physiological resistance model of susceptible infectability and infected infectiousness (each the inverse of resistance). (*c*) If not all encounters are random (e.g. *R =* 0.75), there can be a repelling curve between the basins of attraction for two stable evolutionary outcomes of the evolution of infected gregariousness (red dotted curve, *g_I,_*_rep_), leading to different coevolutionary points. (*d*) The same results hold for physiological resistance except non-random encounters (lower *R*) more strongly select for resistance investment, as seen by a larger region of attraction for low infected infectiousness values. If all encounters are with kin (*R* = 0), the region of attraction becomes larger for (*e*) behavioural resistance or universal for (*f*) physiological resistance. Far left red curves shifted slightly further left in (*e*) and (*f*) for visual clarity.

Both models agree on broad patterns of coevolution. Unsurprisingly, intersections of evolutionarily stable curves (solid blue with solid red) created coevolutionarily stable points (black dots) but if one curve was evolutionarily unstable (dotted red in figure 2*c–e*), the intersection was unstable (hence such red-blue intersections are not marked with dots in figure 2; see electronic supplementary material for details). There is only one coevolutionarily stable point with all random encounters (*R* = 1; figure 2*a,b*); infected hosts become selfish with coevolution favouring maximum tolerance (i.e. minimal resistance) by infected hosts (*g_I,_*_evo_ = 1) and strong resistance by susceptible hosts (low *g_S,_*_evo_; see attached code for proof that selection always favours higher *g_I,_*_evo_ for *R* = 1). Some kin association (*R* < 1; figure 2*c–f*) can lead to bistability between this selfish attractor and an altruistic attractor where infected hosts altruistically invest in resistance (low *g_I,_*_evo_) so susceptible hosts do not need to (high *g_S,_*_evo_). This bistability arises despite a curvature in the costs of resistance that selects for intermediate trait values. Perhaps surprisingly, the altruistic attractor is stable at even weak levels of kin association (e.g. *R* = 0.99, not shown), though weaker kin association does lead to smaller regions of attraction for this altruistic attractor (as seen in figure 2*c,d*; compare to figure 2*e,f*); the surprising feasibility of altruism arises due to the very costs of altruistic resistance by infected hosts. Infected hosts pay a high cost of resistance while susceptible hosts do not. So infected hosts pay a high, direct fitness cost of their investment in resistance but there is also a strong, indirect benefit of investment in resistance; resistance by infected hosts prevents kin from becoming infected and also paying the high costs of resistance borne only by infected hosts. Thus, even weak kin association leads to stability of the altruistic attractor (see electronic supplementary material and figure 2*a* for details).

Selection for infected resistance near the altruistic attractor is so strong that it can select for infected resistance to prevent spreading a mutualist. As a biologically unrealistic example to demonstrate the strength of the mechanism, we changed only the virulence of infection (*v* = −0.048) so that infected hosts live longer than susceptible hosts and kept all other aspects of the model the same. We find selection for stronger infected resistance (lower *g_I,_*_evo_) near the altruistic attractor even if resistance prevents transmission of a mutualist (we used *R* = 0.5 for both models); we found this pattern whether infected hosts reduced the spread of the mutualist behaviourally or physiologically. Our two models display broadly similar patterns of how kin association creates bistability between altruistic and selfish attractors but their main difference is in how strongly kin association drives evolution of resistance.

The models of behavioural and physiological resistance differ in how strongly kin association drives coevolution toward the altruistic attractor. We trace this difference between the two models to terms present in the behavioural resistance model but not the physiological resistance model; these terms represent the cost of resistance investment by non-focal individuals (through fewer social contacts for the focal individual) and these terms maintain the stability of the selfish attractor even with strong kin association (see electronic supplementary material, figure A1). Thus, decreasing the proportion of random encounters leads to evolution only toward the altruistic attractor in the physiological resistance model because the costs of resistance are only borne by the individual rather than by the individual and their kin as in the behavioural resistance model.

**Figure 3.**
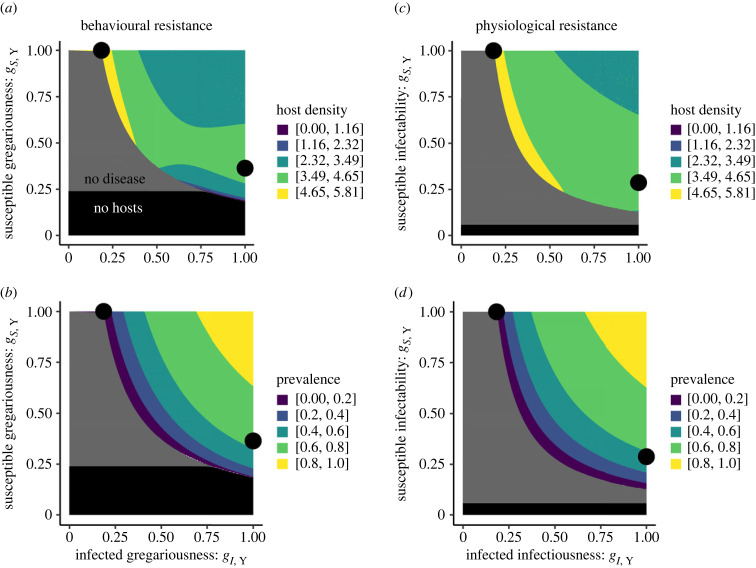
Selfish infected hosts spread parasites and harm host populations while altruistic infected hosts eliminate parasites and boost host populations. We show stable equilibrium host densities (top row, H*) and prevalence of infection (bottom row, p* = I* / H*) for a population that has evolved to some pair of one infected trait value and one susceptible trait value for the behavioural resistance model (left column) and the physiological resistance model (right column). We also use black points to highlight the trait values of coevolutionary outcomes at the altruistic and selfish attractors (values shown for *R* = 0.75 but similar for other values of *R* as long as those attractors exist; figure 2). The dynamics of a population with one genotype for the susceptible host trait and one genotype for the infected host trait do not depend on the value of *R* so that only the location and likelihood of coevolutionary outcomes is affected by *R*.

Both models agree on the broad patterns of how the traits of susceptible and infected hosts combine to determine population-level outcomes for infection prevalence and host density. Host populations have the highest density and lowest infection prevalence when coevolution arrives at the altruistic attractor (resistant infected hosts) rather than the selfish attractor (tolerant infected hosts). Host density is maximized when susceptible hosts have low resistance, enjoying high fitness, while infected hosts have strong resistance/low tolerance/low infectiousness (figure 3*a*). This trait pairing eliminates parasites (the altruistic attractor rests just on the parasite-free boundary, figure 3*b*) so that all hosts enjoy the high fitness of being infection-free without strong resistance investment (because all hosts are susceptible). The selfish attractor instead leads to high prevalence of infection and low density of hosts as the host population is very infected *and* susceptible hosts are forced to maintain strong resistance investment. Results are qualitatively very similar for the physiological resistance model (figure 3*c,d*). Generally, stronger resistance investment by infected hosts increases host density; but the behavioural resistance model differs slightly by demonstrating an exception to this trend. When susceptible gregariousness is low and hosts struggle to find social contacts, higher infected gregariousness (lower resistance) can actually *increase* host density (see bottom right of figure 3*a*). This density pattern only arose for behavioural resistance (not physiological resistance) because the social benefits of gregariousness are shared among hosts. Still, the two models agree strongly overall that resistance investment by infected hosts helps the host population by eliminating parasites, allowing susceptible hosts to omit investment in resistance, and raising population density.

Our key patterns outlined here demonstrated reasonable robustness to parameter values in the parameter sets we searched (±50% for each parameter value except *R* which ranged from 0 to 1). We detected a stable altruistic attractor in 86.7% of feasible parameter sets for the behavioural resistance model and 91.5% for the physiological model. We detected a stable selfish attractor in 70.8% of feasible parameter sets for the behavioural resistance model and only 11.6% for the physiological model, reinforcing our conclusion that kin association drives selection toward the altruistic attractor more strongly in the physiological model (e.g. compare to figure 2*e*,*f* where only the behavioural model has a stable selfish attractor). The altruistic attractor always corresponded to higher host density and the boundary where parasites go to zero while the selfish attractor maintained parasites and lower host density. Thus, our core results may have robust and broad biological relevance.

## 4. Discussion

Considering coevolution of infected and susceptible resistance gives a fuller picture of why selection may favour infected hosts that block transmission. Previous intuition has focused primarily on the selection on either susceptible (for direct fitness) or infected resistance (for kin association), leading to a less complete picture. For example, one could consider a fixed, strong resistance investment by susceptible hosts, thus completely missing the possibility for altruism and finding that lower resistance of infected hosts increases host density (e.g. considering a horizontal slice of figures 2*c* and 3*a* at *g_S_*_, y_ = 0.3). Further, the costs and impacts of resistance investment by susceptible versus infected hosts strongly influence evolution, expanding the possibility for altruism as infection shifts kin from high-fitness, low-resistance susceptible hosts to low-fitness, high-resistance infected hosts. By considering coevolution of infected and susceptible traits, our results emphasize that even weak kin association may be important in models of resistance, with large implications for prevalence and host density.

Future modelling could consider whether metapopulation dynamics expand the biological relevance of the altruistic attractor. If host populations experience stochastic extinction, populations with altruistic traits are favoured for their higher host density, which is empirically associated with less extinction [63] and thus performs better in competition across a metapopulation. Thus, a metapopulation model with stochastic extinction might find even more biological relevance of the altruistic attractor (note, this metapopulation reasoning does not apply to the biologically odd case of a mutualist where infected self-isolation actually reduces host density). The possibility of population extinction will also interact with another future modelling question of interest: pairing our models of host evolution with parasite virulence coevolution, which can also depend on kin association or spatial structure [64], could reveal rich and critical model results. Lastly, future models could consider various impacts of inducibility in behavioural resistance as a difference from physiological resistance. First, some hosts (e.g. meerkats) express their altruistic behaviour without discriminating between kin and non-kin while some express it only for kin [35]. Second and similarly, some susceptible hosts may avoid infecteds specifically [46,65,66] (but see the opposite in [67,68]) while susceptibles may socialize with susceptibles and infected alike in other systems [66,69–71]. Outside the context of kin selection, inducibility has been found to result in some key differences between the evolution of behavioural and physiological resistance [44]; inducibility dependent on kinship and infection status could significantly change the evolution of behavioural resistance compared to physiological resistance. Thus, the intersection of host defence types and kin selection remains a fruitful area for future models.

Our modelling also suggests future empirical work. Very strong versus weak kin association explains why infected hosts become less gregarious in social insects [20,21,27] and infected bacteria kill themselves; in contrast, our modelling predicts that systems with low rates of kin association, as seems likely in our focal system, will have a large basin of attraction for the ‘selfish’ attractor with infected hosts becoming more gregarious. Such increased gregariousness after infection has been observed in sticklebacks [38] and rhesus monkeys [37]. But beyond these simple cases is a broad continuum of the rate of kin association in nature. Our model predicts that even low rates of kin association can potentially select for infected hosts to resist infection instead of tolerating it (bistability between ‘selfish’ and ‘altruistic’ attractors), even when resistance is very costly for direct fitness compared to tolerance. But more systematic, empirical investigation in systems with moderate kin association may reveal evidence for altruistic infected resistance.

For behavioural resistance, empirical evidence could be found as selection for self-isolation by infected hosts that does not improve direct fitness. Determining the mechanism for reduced gregariousness is often difficult as infected hosts may be less mobile, contributing to reduced encounters, or less gregarious given encounter in spatially constrained encounters [72–74]. Whatever the proximate mechanism, if infected hosts have some control over their own social contact rate (e.g. evidenced by heritable variation in the degree that pathogenicity influences sickness behaviour), such an observation could support the prediction of selection for altruistically reduced gregariousness. There is some intriguing evidence for reduced gregariousness of infected hosts outside eusocial insects (e.g. in a mouse system [36]).

Our understanding of the evolution of gregariousness, however, is complicated by under-characterized and potentially confounding direct and indirect effects on fitness. First, the indirect fitness benefits of infected self-isolation through protecting kin are clear but the direct fitness effects of such self-isolation of infected hosts should be considered as a potential confounding factor. Increased activity correlated with a weaker immune response in zebra finches [28] and sick bats ceased grooming all but their close kin [75]. Second the direct fitness benefits of gregariousness are fairly clear but the indirect fitness benefits of gregariousness for kin are less so. Our model assumes that higher gregariousness of a focal individual improves the social component of fitness for a non-focal individual (supported by the impact of group size across many mammalian taxa [6]); but clear, empirical quantification of both direct and indirect fitness effects would strongly reinforce our model conclusions and inspire further work comparing and contrasting physiological and behavioural host defences. Disentangling as many of the possible direct and indirect fitness effects of gregariousness will improve future investigations of gregariousness, infection, and kin selection across systems.

For physiological resistance, investment in resistance rather than tolerance could indicate altruism by infected hosts. Resistance that controls the number of parasites on an infected host reduces transmission to conspecifics [76] but may trade-off with tolerance of those parasites [16–19]. Resistance may improve the fitness of an infected host by decreasing parasite burdens or worsen fitness if the sacrifice in tolerance matters more than the reduction in parasite burden (as depicted in figure 1*a*,*b*). Such resistance that worsens infection outcomes may be an indication of altruism by infected hosts and could be investigated by future experiments in systems with weaker kin association than clonal bacteria [30]. All together, these modelling results emphasize that altruism by infected hosts may be more relevant than previously thought, emphasizing the importance of coevolution of infected and susceptible traits both for behavioural and physiological resistance.

## Data Availability

All codes required to reproduce these analyses are provided as electronic supplementary material [77].
